# Supplementary material for: An Emerging Strategy for Muscle Evanescent Trauma Discrimination by Spectroscopy and Chemometrics
Source: Int J Mol Sci. 2022 Nov 4;23(21):13489. doi: 10.3390/ijms232113489 (PMC9658611; doi:10.3390/ijms232113489)
Supplement: Supplementary file 1 [file ijms-23-13489-s001.zip › ijms-1939965-supplementary.pdf]

International Journal of
Molecular Sciences

MDPI

Article

An Emerging Strategy for Muscle Evanescent Trauma Discrimination by Spectroscopy and Chemometrics

Gongji Wang, Hao Wu, Canyu Yang, Zefeng Li, Run Chen, Xinggong Liang, Kai Yu, Huiyu Li, Chen Shen, Ruina Liu, Xin Wei, Qinru Sun, Kai Zhang * and Zhenyuan Wang *

Department of Forensic Pathology, College of Forensic Medicine, Xi'an Jiaotong University, Xi'an 710061, China

* Correspondence: zkzjly@xjtu.edu.cn (K.Z.); wzy218@xjtu.edu.cn (Z.W.)

Citation: Wang, G.; Wu, H.; Yang, C.; Li, Z.; Chen, R.; Liang, X.; Yu, K.; Li, H.; Shen, C.; Liu, R.; et al. An Emerging Strategy for Muscle Evanescent Trauma Discrimination by Spectroscopy and Chemometrics. *Int. J. Mol. Sci.* **2022**, *23*, 13489. https://doi.org/10.3390/ijms232113489

Academic Editor: Małgorzata Barańska

Received: 13 September 2022
Accepted: 2 November 2022
Published: 4 November 2022

Publisher's Note: MDPI stays neutral with regard to jurisdictional claims in published maps and institutional affiliations.

Copyright: © 2022 by the authors. Licensee MDPI, Basel, Switzerland. This article is an open access article distributed under the terms and conditions of the Creative Commons Attribution (CC BY) license (https://creativecommons.org/licenses/by/4.0/).

Supplementary materials

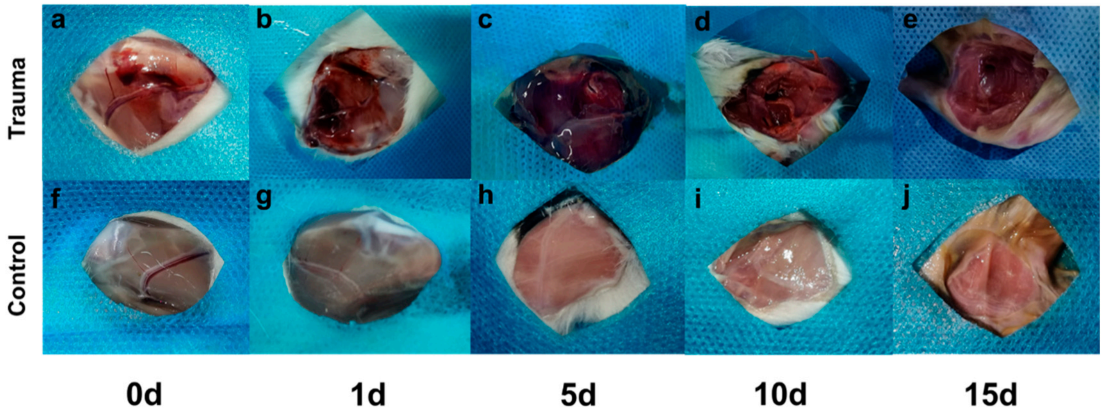

Figure S1. Macroscopic visualization and temporal changes of muscle trauma group and control group. a, f: macroscopic display of rat muscle trauma group and control group in 0d; b, g: macroscopic display of rat muscle trauma group and control group in 1d; c, h: macroscopic display of rat muscle trauma group and the control group in 5d; d, i: macroscopic display of rat muscle trauma group and control group in 10d; e, j: Macroscopic display of rat muscle trauma group and control group in 15d.

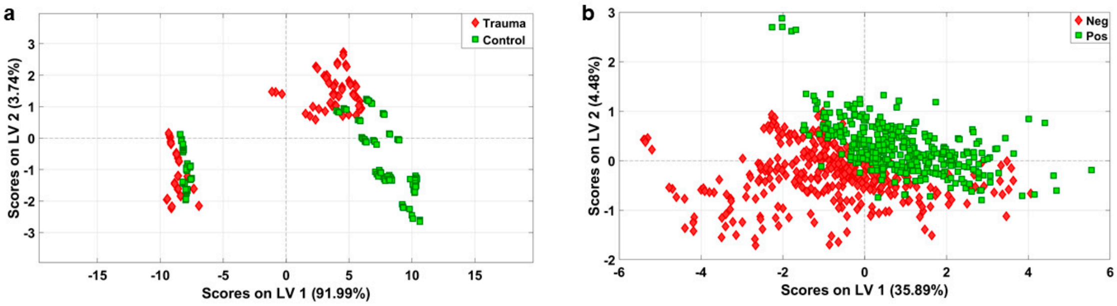

Figure S2. PLS-DA analysis scores plots for rat and human sample datasets. a: Rat muscle sample dataset PLS-DA analysis score graph; b: Human muscle sample dataset PLS-DA analysis score graph.

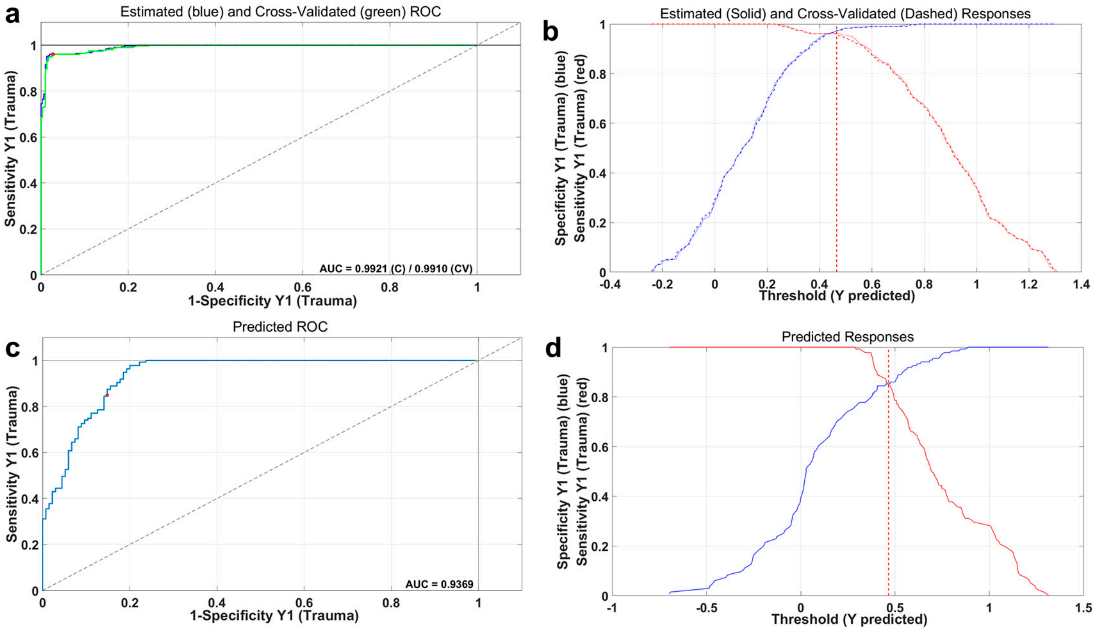

Figure S3. ROC curve of rat muscle trauma recognition model and response map of related indexes. a: ROC curve of the model established by the rat muscle training set and the corresponding AUC value. b: Responses of sensitivity and specificity of the model established from the rat muscle training dataset. c: ROC curve and corresponding AUC values of the model predicted by the rat muscle external validation data set. d: Responses of sensitivity and specificity of rat muscle external validation dataset to model prediction.

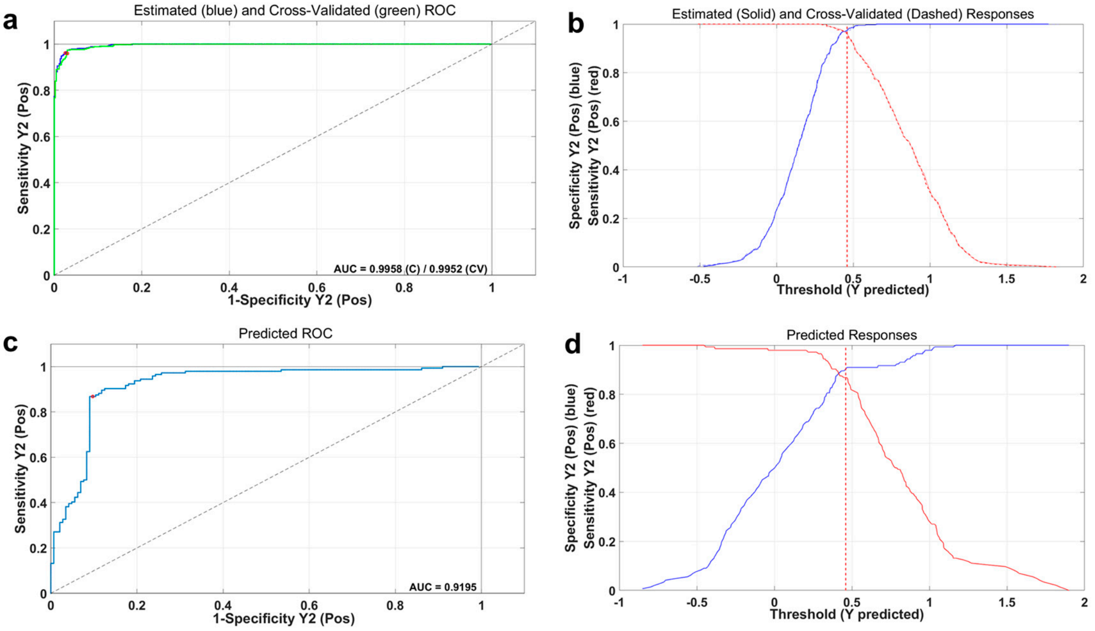

Figure S4. ROC curve of human muscle trauma recognition model and response map of related indexes. a: ROC curve of the model established by the human muscle training set and the corresponding AUC value. b: Responses of sensitivity and specificity of the model established from the human muscle training dataset. c: ROC curve and corresponding AUC values of the model predicted by the human muscle external validation data set. d: Responses of sensitivity and specificity of human muscle external validation dataset to model prediction.

a

Number PCs: 8 Auto Select

Percent Variance Captured by PCA Model (* = suggested)

	Eigenvalue of Cov(X)	% Variance This PC	% Variance Cumulative	RMSEC	RMSECV
1	5.38e+01	93.78	93.78	0.08737	0.09576
2	1.61e+00	2.81	96.59	0.06471	0.07484
3	5.92e-01	1.03	97.62	0.05405	0.06994
4	4.65e-01	0.81	98.43	0.04391	0.06025
5	2.79e-01	0.49	98.92	0.03649	0.05192
6	1.55e-01	0.27	99.19	0.03162	0.04822
7	9.30e-02	0.16	99.35	0.02831	0.0452
8	8.55e-02	0.15	99.50	0.02487	0.04187
9	5.69e-02	0.10	99.60	0.02229	0.04035
10	4.90e-02	0.09	99.68	0.0198	0.03894
11	3.74e-02	0.07	99.75	0.01767	0.03814
12	2.78e-02	0.05	99.79	0.0159	0.03645
13	2.38e-02	0.04	99.84	0.01421	0.03532
14	1.86e-02	0.03	99.87	0.01274	0.03397
15	1.37e-02	0.02	99.89	0.01153	0.03263
16	1.13e-02	0.02	99.91	0.01044	0.03213
17	1.01e-02	0.02	99.93	0.009347	0.03027
18	8.20e-03	0.01	99.94	0.008358	0.02893
19	5.37e-03	0.01	99.95	0.00764	0.02853
20	4.75e-03	0.01	99.96	0.006943	0.02821

b

Number PCs: 6 Auto Select

Percent Variance Captured by PCA Model (* = suggested)

	Eigenvalue of Cov(X)	% Variance This PC	% Variance Cumulative	RMSEC	RMSECV
1	2.92e+00	36.24	36.24	0.104	0.1217
2	2.76e+00	34.35	70.59	0.07061	0.08204
3	7.71e-01	9.58	80.17	0.05797	0.07378
4	5.08e-01	6.31	86.49	0.04786	0.06506
5	3.43e-01	4.26	90.75	0.0396	0.05874
6	2.87e-01	3.57	94.32	0.03104	0.04911
7	1.29e-01	1.61	95.92	0.02629	0.04937
8	9.48e-02	1.18	97.10	0.02217	0.04592
9	5.49e-02	0.68	97.78	0.01939	0.04046
10	4.65e-02	0.58	98.36	0.01667	0.03858
11	3.75e-02	0.47	98.83	0.0141	0.03562
12	2.27e-02	0.28	99.11	0.01229	0.03595
13	1.65e-02	0.21	99.31	0.01078	0.03495
14	1.21e-02	0.15	99.46	0.009526	0.03614
15	9.90e-03	0.12	99.59	0.00836	0.03456
16	7.17e-03	0.09	99.68	0.007401	0.03211
17	6.82e-03	0.08	99.76	0.006357	0.02878
18	3.17e-03	0.04	99.80	0.005807	0.02838
19	2.49e-03	0.03	99.83	0.005337	0.02742
20	2.04e-03	0.03	99.86	0.004917	0.02674

Figure S5. RMSEC and RMSECV for PCA analysis of rat and human datasets. a: RMSEC and RMSECV for PCA analysis of rat datasets; b: RMSEC and RMSECV for PCA analysis of human datasets.
